# Engineering Self-Assembled PEEK Scaffolds with Marine-Derived Exosomes and Bacteria-Targeting Aptamers for Enhanced Antibacterial Functions

**DOI:** 10.3390/jfb17010023

**Published:** 2025-12-30

**Authors:** Chen Zhang, Jinchao You, Runyi Lin, Yuansong Ye, Chuchu Cheng, Haopeng Wang, Dejing Li, Junxiang Wang, Shan Chen

**Affiliations:** 1 School of Materials and Chemistry Engineering, College of Geography and Oceanography, Minjiang University, Fuzhou 350108, China; zhangchen@mju.edu.cn (C.Z.); yeyuansong@mju.edu.cn (Y.Y.); lidejing@mju.edu.cn (D.L.); 2 Fuzhou Institute of Oceanography, Minjiang University, Fuzhou 350108, China; 3 School of Public Health, Fujian Medical University, Fuzhou 350108, China; youjinchao@fjmu.edu.cn; 4 School of Pharmaceutical Sciences, Jilin University, Changchun 130021, China; linry25@mails.jlu.edu.cn; 5 Engineering Training Center, Minjiang University, Fuzhou 350108, China; chuchucheng@mju.edu.cn; 6 CAS Key Laboratory of Design and Assembly of Functional Nanostructures, Fujian Key Laboratory of Nanomaterials, Fujian Institute of Research on the Structure of Matter, Chinese Academy of Sciences, Fuzhou 350002, China; wanghaopeng@fjirsm.ac.cn

**Keywords:** 3D printing, PEEK scaffold, aptamer, marine-derived exosome, antibacterial

## Abstract

Repairing bone defects with implants is an important topic in the field of regenerative medicine, but bacterial infection presents a significant barrier in clinical practice. Therefore, bone implants with antibacterial functionality are currently in high demand. Fresh seaweed-derived exosomes (EXOs) exhibited promising antibacterial activity against bacteria, indicating their potential as natural antimicrobial agents. Moreover, equipping the exosomal lipid bilayer with bacteria-targeting aptamers (Apt), termed EXOs-Apt, enabled precise bacterial killing, thereby promoting more effective antibacterial functions. In our design, porous polyetheretherketone (PEEK) scaffolds were 3D-printed using the melt deposition manufacturing process. Subsequently, the scaffold surfaces were modified via dopamine self-polymerization, resulting in the formation of a polydopamine (PDA) coating. Then, EXOs-Apt was applied to functionalize PEEK scaffolds with antibacterial activity. Given that EXOs display bactericidal effects while Apt facilitates bacterial capture, we engineered a surface coating platform that incorporates both components to produce a multifunctional scaffold with synergistic antibacterial activity. The results showed that modifying EXOs-Apt on PEEK scaffolds significantly improved their antibacterial performance against *Escherichia coli* and *Staphylococcus aureus*. To our knowledge, this is the first study to use EXOs-Apt as antibacterial coatings modified on PEEK scaffolds. This study provides new strategies and ideas for the development of antibacterial PEEK orthopedic implants with promising clinical value for infection-resistant repair of bone defects.

## 1. Introduction

As one of the global challenges in orthopedic treatment, bone defects involve multiple fields such as materials science, engineering, and regenerative medicine. With the increasing aging of the population and the rising incidence of large bone defects caused by bone tumors, osteomyelitis, sports injuries, congenital malformations, and traumatic accidents [1,2,3,4,5], there is currently a substantial clinical demand for bone implants [6]. Particularly, infections are common in transplant surgeries: once an implant infection occurs, the implant needs to be removed, leading to the failure of the transplant surgery. In severe cases, amputation surgery may even be necessary to avoid life-threatening situations. At present, research generally believes that implant infection is caused by bacteria adhering to the surface of the implant and gradually proliferating to form a bacterial membrane [7,8]. Therefore, seeking suitable bone tissue substitutes has become a thorny issue in the field of bone repair.

PEEK is a thermoplastic polymer material synthesized through condensation reactions. The rigid benzene ring, flexible ether bonds, and carbonyl groups that enhance intermolecular interactions collectively produce PEEK’s unique structural organization. This structural characteristic not only endows PEEK with excellent mechanical properties, but also confers additional advantages, such as high temperature resistance, hydrolysis resistance, wear resistance, chemical corrosion resistance, and flame retardancy. Additionally, it exhibits good biocompatibility, radiation transparency, and does not produce artifacts in nuclear magnetic resonance [9]. Thanks to these outstanding properties, PEEK was certified by the U.S. Food and Drug Administration (FDA) in the 1980s and approved for use in the manufacture of orthopedic implants. After the 1990s, it gradually developed into a key alternative material for metal implants and has been widely used in treatment fields such as fracture repair and joint replacement [10,11]. Advancements in materials science have led to the development of carbon fiber-reinforced PEEK composites, further broadening their potential for bone repair and other medical applications [4,12,13,14].

3D printing technology is an advanced personalized manufacturing process that operates by following computer-generated instructions to melt materials and layer them based on a digital 3D model, resulting in the fabrication of a solid object [15,16]. Its capacity to produce intricate models with minimal material waste, coupled with exceptional molding efficiency and flexible design possibilities, significantly outperforms traditional production techniques [17,18,19,20,21]. Fused Deposition Modeling (FDM) is a widely used 3D printing process that can reach temperatures of up to 400 °C and is mainly used for printing thermoplastic materials. Therefore, in the field of medical implant manufacturing, the integration of FDM with PEEK has emerged as a prominent research direction for the patient-specific production of bone implants [22,23].

Although PEEK has many excellent properties suitable for use as bone implants, its clinical application is limited due to its biological inertness [24,25]. Experimental data show that PEEK materials suffer from poor antibacterial performance and limited bone tissue integration during implantation [26,27,28]. These problems not only hinder effective implant–bone tissue integration, but also elevate the risk of bacterial infections and the onset of infectious complications [10]. Currently, the strategies for improving the antibacterial and osseointegration capabilities of PEEK material implants mainly focus on material composites and surface modification. In terms of material composites, researchers typically combine bioactive substances such as hydroxyapatite (HA) with a PEEK matrix to enhance its biological properties [29,30]. However, this method has obvious limitations, as the introduction of new substances alters the original interfacial bonding energy of PEEK, typically reducing the mechanical strength of the composite and potentially shortening the implant’s service life [31,32]. Compared to other methods, surface modification technology improves the biological function of the PEEK matrix by regulating its surface morphology and chemical properties. It not only maintains the excellent mechanical properties of the PEEK matrix, but also enhances surface antibacterial properties in a targeted manner [28]. Therefore, it is considered as a more promising optimization solution.

Marine macroalgae-derived exosomes (EXOs) exhibit unique potential in the field of antibacterial activity [33,34]. Their antibacterial function is mainly achieved through two pathways: direct inhibition of pathogenic microorganisms and regulation of host immunity. A recent study demonstrated that polyphenol-engineered exosomes derived from *Laminaria japonica* effectively inhibit the formation of advanced glycation end products and reverse the inflammatory microenvironment, thereby maintaining antibacterial activity under high-glucose conditions [35]. In addition, other exosomes derived from marine sources also process the ability to carry antibacterial molecules, such as specific miRNAs or proteins, which can be delivered to the site of infection. This delivery can directly interfere with bacterial growth or disrupt the formation of bacterial biofilms. At the same time, these exosomes can enhance the body’s immune clearance mechanisms by modulating macrophage polarization and promoting the release of pro-inflammatory cytokines [36,37,38,39,40]. These characteristics underscore the significant translational potential of marine-derived exosomes in combating bacterial infections.

Aptamer (Apt) is single-stranded DNA or RNA molecules typically obtained by systematic evolution of ligands by exponential enrichment (SELEX), and can bind bacterial surface markers such as membrane proteins and lipopolysaccharides with high affinity and specificity [14,41,42,43]. Given the advantages of high thermal stability, easy chemical modification, and low immunogenicity, Apt has attracted substantial attention as versatile molecular tools for bacterial capture [4,44]. Modifying Apt on the surface of PEEK material with antibacterial EXOs could yield synergistic antibacterial activity and substantially improve antibacterial performance.

**Figure 1:**
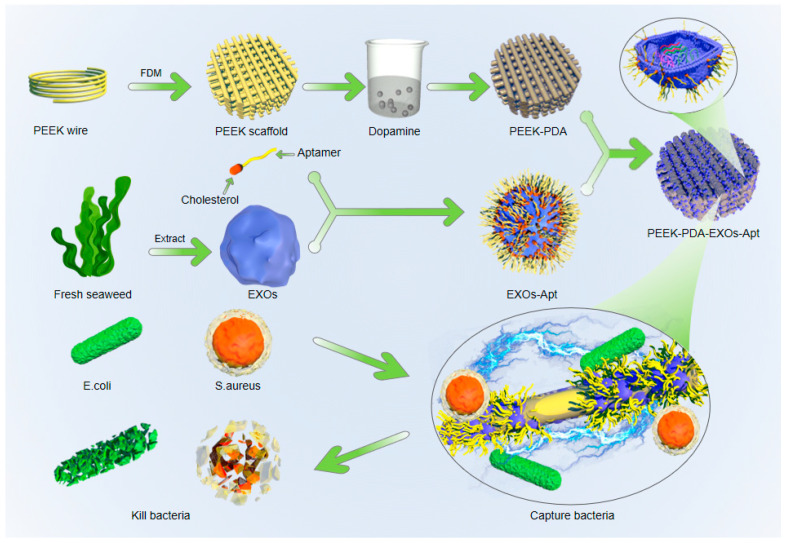
Schematic illustration of self-assembled PEEK scaffolds with marine-derived exosomes and bacteria-targeting aptamers for enhanced antibacterial functions.

The aim of this study was to prepare PEEK implant scaffolds that combine mechanical robustness with antibacterial activity through a green and convenient method. We employed FDM technology with PEEK as the printing material to create a porous scaffold model for orthopedic implants (Figure 1). Dopamine self-polymerization was used to prepare a PDA coating on the surface of PEEK scaffolds. Cholesterol-conjugated aptamers were inserted into the exosomal lipid bilayer via hydrophobic interactions, thereby stably anchoring the aptamers on the EXOs surface. Finally, EXOs-Apt were modified onto PEEK-PDA using electrostatic adsorption. As a result, EXOs-Apt on PEEK scaffolds significantly improved their antibacterial performance against *Escherichia coli* and *Staphylococcus aureus*, offering a promising approach to develop PEEK orthopedic implants with enhanced antibacterial functions.

## 2. Results

### 2.1. Morphology Observations

**Figure 2:**
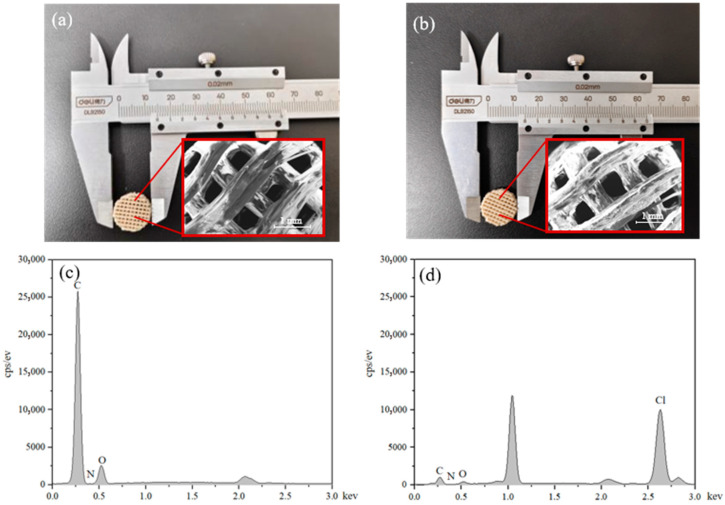
Macroscopic morphology, SEM and EDS of scaffolds (Scale bar 1 mm). (**a**) Photograph and SEM of pure PEEK scaffold; (**b**) Photograph and SEM of PEEK-PDA scaffold; (**c**) EDS of pure PEEK scaffold; (**d**) EDS of PEEK-PDA scaffold.

The porosity of bone tissue refers to the percentage of the volume of internal pores in bone tissue to the total volume of bone tissue. Both large and small pore sizes are not conducive to cell adhesion. Artificial bone scaffolds with a porosity range of 30% to 90% have a porosity comparable to that of human bone trabeculae. Therefore, artificial bone scaffolds with a porosity range within this range are considered the best. In this study, a 50% porosity model was prepared as the scaffold, with a diameter of 15 mm and a height of 5 mm. The 3D-printed scaffolds have uniform macro morphology. After modification with dopamine hydrochloride, the surface of the scaffold was immobilized with PDA and the color became darker (Figure 2a,b). Table S1 displays the measurement values of height, diameter, and weight for each group of scaffolds. Pure PEEK contains a relatively high proportion of oxygen and carbon elements (Figure 2c), while the observed increase in chlorine content demonstrates that PEEK was successfully modified with PDA (Figure 2d).

### 2.2. SEM Characterization of Scaffolds

**Figure 3:**
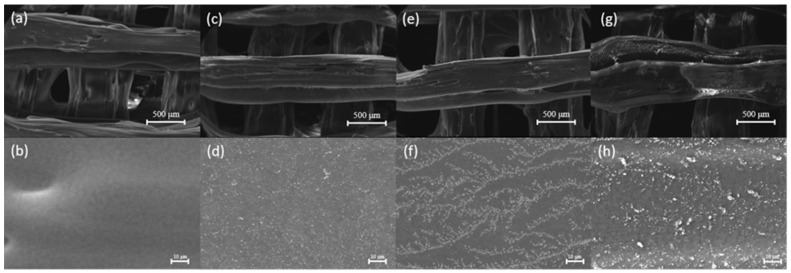
SEM characterization images of scaffolds (scale bar 500 μm; 10 μm). (**a**,**b**) pure PEEK scaffolds; (**c**,**d**) PEEK-PDA scaffold; (**e**,**f**) PEEK-PDA-EXOs scaffold; (**g**,**h**) PEEK-PDA-EXOs-Apt scaffold.

As shown in Figure 3a,b, the surface of pure PEEK is clean and free of other particles. In Figure 3c,d, a dense PDA coating can be observed on the surface of the scaffold. This modification provides a foundation for further functionalization of the stent surface. In Figure 3g,h, the morphological characteristics of the final coating can be seen. Due to further modification, the surface appearance of the coating has changed compared to Figure 3e,f.

### 2.3. Antibacterial Activity of EXOs

**Figure 4:**
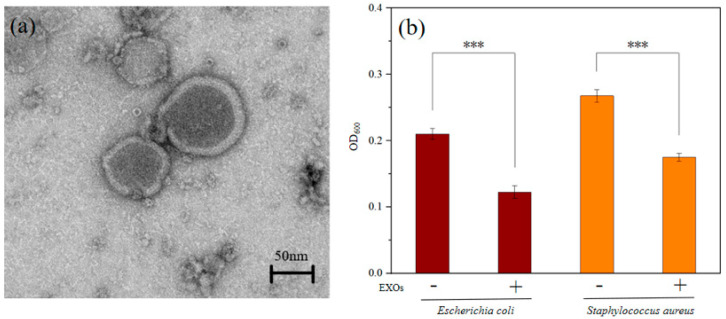
The characteristic and antibacterial test of EXOs. (**a**) TEM image of EXOs (scale bar 50 nm); (**b**) Antibacterial effect of EXOs on *Escherichia coli* and *Staphylococcus aureus* (*** *p* < 0.001).

We employed transmission electron microscopy to characterize the morphology of fresh seaweed-derived EXOs. The result showed that the purified EXOs had a uniform morphology and a distinct bilayer membrane structure on the outside (Figure 4a). Next, we further verified the antibacterial functions of EXOs. As shown in Figure 4b, after 12 h of cultivation, according to the antibacterial rate (%) = [(OD_600blank_ − OD_600experimental group_)/OD_600blank_] × 100%, the antibacterial rate of EXOs against *Escherichia coli* can be calculated as 39.05%, and the antibacterial rate against *Staphylococcus aureus* was 15.38%. The data shows that EXOs have a good antibacterial effect on both *Escherichia coli* and *Staphylococcus aureus*.

### 2.4. Antibacterial Performance of Modified Scaffolds

**Figure 5:**
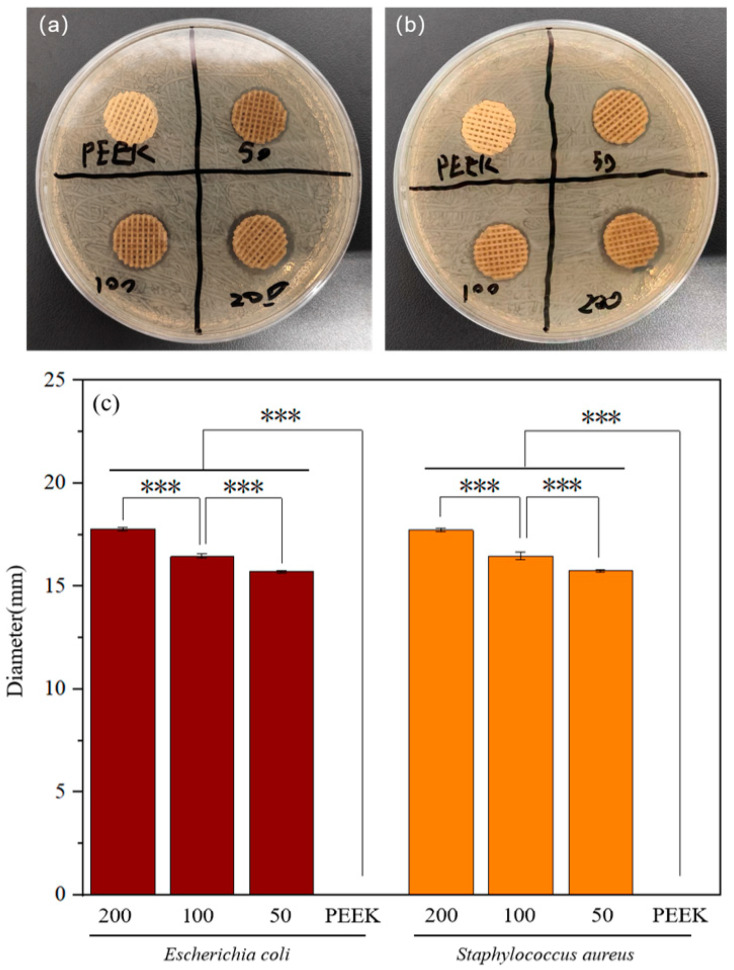
Photographs of (**a**) *Escherichia coli* and (**b**) *Staphylococcus aureus* inhibition zones of scaffolds. (**c**) Significance analysis chart of inhibition zones (PEEK represents the pure PEEK group, while 50, 100, and 200 represent the experimental groups of Apt modified scaffolds with concentrations of PEEK-PDA-EXOs-Apt 50 nM, PEEK-PDA-EXOs-Apt 100 nM, and PEEK-PDA-EXOs-Apt 200 nM, respectively, *** *p* < 0.001).

To investigate the effect of aptamer concentration on the antibacterial performance of the scaffolds, we measured the diameter of the antibacterial zones around the scaffolds on LB agar plates inoculated with *Escherichia coli* and *Staphylococcus aureus*, respectively (Figure 5a,b). We recorded the diameters of inhibition zones as shown in Table S2. As shown in Figure 5c, with the increase in Apt concentration, the inhibitory effect of the scaffold on Gram-positive bacteria and Gram-negative bacteria gradually strengthened.

## 3. Discussion

In this study, we demonstrated that the EXOs extracted from seaweed had antibacterial properties. After modification with Apt, the antibacterial effect significantly improved, and the extent of the improvement in the antibacterial effect was proportional to the amount of Apt used. Here, we utilized the electrostatic adsorption of PDA to modify EXOs-Apt on PEEK scaffolds, improving the surface antibacterial performance of PEEK scaffold and providing a new strategy for preparing antibacterial bone implants.

The porosity and pore size of artificial bone implants should be similar to natural bone; pores and pore sizes beyond a certain range are not conducive to bone tissue adhesion and growth [4,45,46]. The performance of implants is optimal when the porosity range is between 30% and 90%, and a consistent pore structure is necessary for tissue cells to grow inward during bone tissue regeneration [45,47]. In this study, a 50 w% porosity model was selected to prepare the scaffold. As shown in Figure 2a,b, by using staggered 3D printing linear arrangement, a scaffold with uniform pores was prepared, providing a suitable three-dimensional growth space for tissue cell proliferation and optimizing biological performance. As shown in Table S1, the standard deviation of each group of scaffolds was less than 0.3, indicating that the PEEK scaffolds prepared in this study had small fluctuations in size and weight values, and the samples prepared using this preparation method had good repeatability.

Dopamine (DA) is considered a suitable substitute for natural materials due to its catechol and amine functions [48]. DA hydrochloride can self-polymerize into PDA in an alkaline environment, which has been proven to have strong binding ability for many substances, including EXOs [49,50,51,52,53]. Comparing Figure 2a and Figure 2b, the color of the PEEK scaffold darkened after modifying PDA. The observed increase in chlorine content in Figure 2d further confirms the successful modification of PEEK by PDA. EXOs-Apt has already been modified on the surface of scaffolds, which is crucial for improving the biological performance of the implant.

Several compounds in the ocean have been identified as potential antimicrobial agents, including alkaloids extracted from sponges, polysaccharides extracted from marine crustaceans, and cellulose extracted from seaweed [54,55,56]. Here, we extracted EXOs from seaweed and determined whether EXOs have antibacterial activity against *Escherichia coli* and *Staphylococcus aureus*, respectively, and how active they are by observing changes in the OD_600_ of the bacterial solution. The antibacterial pre-experiment of EXOs showed that the increase rate of OD_600_ in the bacterial solution with EXOs added was slower than that in the pure bacterial solutions, indicating that EXOs have an inhibitory effect on bacteria. The differential antibacterial activity of EXOs against *Escherichia coli* (39.05%) and *Staphylococcus aureus* (15.38%) suggests that the antibacterial mechanism may be more effective against Gram-negative bacteria. This could be related to differences in cell wall structure between Gram-positive and Gram-negative bacteria.

It is worth mentioning that we added Apt to the coating material, which has been proven to have the ability to capture bacteria [57,58,59]. We modified liposomes on Apt and used hydrophobic interactions to insert Apt onto EXOs, thereby combining the bacterial recruitment function of Apt with the antibacterial function of EXO to achieve a combined antibacterial effect. We hope to improve the antibacterial performance of the coating. As shown in Figure 5c, with the increase in Apt concentration, the antibacterial zone of the scaffold gradually increased, indicating that the combined antibacterial effect of EXOs-Apt significantly improves the antibacterial effect of the coating. This provides new ideas for the study of the synergistic antibacterial activity of biomolecules.

## 4. Materials and Methods

### 4.1. Preparation of PEEK Scaffolds and PDA Modification

Porous PEEK scaffolds were designed with a diameter of 15 mm, a height of 5 mm, and a target porosity of 50%. The scaffolds were fabricated using an FDM 3D printer (Shenzhen Kuaizao Technology Co., Ltd., Shenzhen, China) and PEEK wires (Solvay Group, Brussels, Belgium). The printing process was optimized with a nozzle temperature of 400 °C and a build plate temperature of 100 °C. To remove surface impurities post-printing, the scaffolds were cleaned sequentially with acetone, anhydrous ethanol (Aladdin Biochemical Technology Co., Ltd., Shanghai, China), and ultrapure water via ultrasonication. Finally, the scaffolds were dried under a nitrogen stream and stored for subsequent use.

The surface of the PEEK scaffolds was modified using a dopamine oxidative self-polymerization method. A dopamine hydrochloride solution (0.5 mg/mL) was prepared by dissolving 5 mg of dopamine hydrochloride (Aladdin Biochemical Technology Co., Ltd., Shanghai, China) in 10 mL of phosphate-buffered saline (PBS; Thermo Fisher Scientific, Shanghai, China). The pH of the buffer was adjusted to 8.5 using a sodium hydroxide solution to trigger polymerization. The PEEK scaffolds were immersed in the dopamine solution for 4 h, allowing for the formation of a PDA coating on the scaffold surfaces. Following the modification, the scaffolds (designated as PEEK-PDA) were removed, rinsed three times with ultrapure water under ultrasonication to eliminate residual monomers, and dried in a vacuum oven.

### 4.2. Isolation and Characterization of EXOs

Fresh seaweed was thoroughly washed, and the blade-like structures were removed. After recording the wet weight of the seaweed stems, the tissue was homogenized in PBS using a high-speed blender. The resulting homogenate was filtered through gauze to remove bulk mucus and fibers. To isolate EXOs, a differential centrifugation protocol was employed: The filtrate was centrifuged at 1000× *g* for 20 min to remove protoplasts, followed by 5000× *g* for 40 min to eliminate dead cells. The supernatant was further centrifuged at 10,000× *g* for 60 min to remove cellular debris. The resulting supernatant was passed through a 0.22 μm filter membrane to ensure the removal of larger particles and microbial contaminants. The filtrate underwent ultracentrifugation at 100,000× *g* for 1 h to pellet the EVs. The pellet was resuspended in PBS and subjected to a second ultracentrifugation cycle (100,000× *g*, 1 h) to remove residual proteins and impurities. The final EXOs were resuspended in PBS for further analysis.

The morphology of the isolated EXOs was observed using Transmission Electron Microscopy (TEM). Briefly, 5–10 μL of the EXO suspension (pre-fixed with 2.5% glutaraldehyde) was loaded onto a Formvar-carbon coated copper grid and allowed to adsorb at room temperature for 5 min. Excess liquid was carefully removed using filter paper. For negative staining, 10 μL of saturated uranyl acetate solution was applied to the grid for 1 min. After removing the excess stain, the grid was washed twice by adding drops of double-distilled water and allowed to sit for 5 min each. After air-drying at room temperature, the samples were imaged using a TEM (Talos F200s, Thermo Fisher Scientific, Waltham, MA, USA) operating at an accelerating voltage of 80 kV.

### 4.3. Preparation of PEEK Scaffolds Loaded with Antibacterial Coatings (PEEK-PDA-EXOs-Apt)

Firstly, we prepared 0.1 nmol/μL of Apt stock solution (DNA sequence: 5′-Cholesterol-TTTGGGACAGGGAGTGCGCTGCTCTTTTTCGC-3′). The extracted EXOs were diluted with PBS buffer to prepare 0.3 μg/μL of EXO stock solution. Then, EXOs were mixed with the above Apt stock solution at varying ratios, permitting the Apt’s terminal cholesterol moiety to be inserted into the EXOs’s membrane via hydrophobic interactions. The mixture was adjusted to a final volume of 5 mL with PBS. After that, we immersed the PEEK-PDA into the EXOs-Apt solutions described above and allowed them to incubate for 12 h. During this process, due to electrostatic adsorption, the phosphate skeleton (negatively charged) and membrane phospholipids (negatively charged) in EXOs-Apt attract the positively charged amino groups in PDA, thereby modifying EXOs-Apt on the surface of the scaffold. Subsequently, the PEEK scaffolds were removed and ultrasonically cleaned three times with ultrapure water to remove residues. Finally, it was dried in a vacuum drying oven to obtain PEEK scaffolds modified with antibacterial coatings of different concentrations. According to different Apt concentrations, these were called PEEK-PDA-EXOs-Apt50nM, PEEK-PDA-EXOs-Apt100nM, and PEEK-PDA-EXOs-Apt200nM. The PEEK-PDA was immersed in a pure EXOs system (25 μL EXOs stock solution, PBS to 5 mL) for 12 h to prepare a PEEK-PDA-EXO control group.

### 4.4. Morphological Characterization of Modified Scaffolds

The diameter and height of the scaffolds were measured using a vernier caliper, while the weight was measured using a precision balance and the average value calculated for subsequent analysis. In addition, the microstructure characteristics of the scaffold were observed using a scanning electron microscope (SEM, E-1045, HI TACHI; Tokyo, Japan). EDS was used to analyze the modification of the scaffolds and the elemental composition of the scaffolds’ surface.

### 4.5. Examination Antibacterial Activity of EXOs via Absorption Photometry

The antibacterial potential of EXOs was evaluated against *Staphylococcus aureus* and *Escherichia coli* (Ochikohua Medical Supply Chain Management, Tianjin, China). Bacterial strains were cultured overnight to reach the exponential growth phase. The optical density at 600 nm (OD_600_) was measured using a spectrophotometer (Shanghai Yulong Instrument Co., Ltd., Shanghai, China) and adjusted to 0.1 with fresh culture medium. For the assay, 29 μL of the adjusted bacterial suspension was seeded into a 384-well plate. Subsequently, 1 μL of EXOs solution (0.3 μg/μL) was added to each well. A blank control group was prepared by using 30 μL of the bacterial suspension without EXOs. Each group was tested in five parallels. The plates were sealed with a breathable film and incubated at 37 °C for 12 h. The OD_600_ values were then recorded to calculate the antibacterial efficacy.

### 4.6. Absorption Method for Testing Antibacterial Activity

The antibacterial efficacy of the functionalized scaffolds was assessed using the absorption method. The experimental groups included PEEK-PDA-EXOs and PEEK-PDA-EXOs-Apt (prepared with three concentration gradients), while pure PEEK scaffolds served as the control group. Prior to testing, all scaffolds were sterilized by ultrasonication in 75% ethanol for 5 min, rinsed with sterile water, and dried under ultraviolet light in a clean bench for 20 min. *Staphylococcus aureus* and *Escherichia coli* suspensions were prepared at an OD_600_ of 0.1. Each scaffold was placed onto the inner cap of a sterile 50 mL centrifuge tube. A 0.2 mL aliquot of the bacterial suspension was precisely inoculated onto the scaffold surface, ensuring complete absorption without contact with the tube walls. After sealing, the tubes were incubated at 37 °C for 24 h to allow for co-culture. Subsequently, 20 mL of Luria-Bertani broth was added to each tube. The tubes were shaken gently to elute the surviving bacteria from the scaffolds. The OD_600_ of the eluent was measured in triplicate for each sample to determine the average bacterial concentration and evaluate the antimicrobial performance of the modified surfaces.

### 4.7. Antibacterial Zone Test

The formation of inhibition zones was evaluated using the spread-plate method to qualitatively assess the antibacterial properties of the scaffolds. Under sterile conditions in a laminar flow hood, bacterial suspensions of *Staphylococcus aureus* and *Escherichia coli* (OD_600_ = 0.1) were inoculated onto Luria-Bertani agar plates. A sterile spreading rod was used to ensure the uniform distribution of the bacteria across the agar surface. Four groups of scaffolds, comprising pure PEEK-PDA and PEEK-PDA-EXOs-Apt modified with three varying concentrations of Apt, were placed at the center of the inoculated plates. The plates were then incubated at 37 °C for 24 h. Following incubation, the presence of a bacteriostatic zone surrounding each scaffold was observed. The diameter and transparency of these zones were measured and recorded to compare the relative antibacterial efficacy of the different surface modifications.

## 5. Conclusions

In this study, PDA self-polymerized on the surface of the PEEK scaffold as a medium modified with other antibacterial substances. Cholesterol-modified Apt was inserted into EXOs through hydrophobic interactions, yielding EXOs–Apt, which were then modified on the scaffold due to electrostatic adsorption with PDA. The preparation method of this study is environmentally friendly. The macroscopic morphology and SEM images of the scaffold revealed a uniform pore distribution. The SEM-EDS analysis of the bracket confirmed the successful coating modification. The antibacterial effects of EXOs and the high-affinity bacteria capture of Apt synergistically enhance antibacterial activity. The optimal effect was observed when the Apt concentration reached 200 nM. The scaffold demonstrated significant antibacterial effects against *Escherichia coli* and *Staphylococcus aureus*, highlighting its potential as a promising implant material for the treatment of bone defects.

## Data Availability

The original contributions presented in the study are included in the article, further inquiries can be directed to the corresponding author.

## Supplementary Materials

The following supporting information can be downloaded at: https://www.mdpi.com/article/10.3390/jfb17010023/s1, Table S1: Measurement results of weight, diameter, and height of composite scaffolds, Table S2: Size of the antibacterial zone of the scaffolds.

## Abbreviations

The following abbreviations are used in this manuscript:

EXOs	Exosomes
Apt	Aptamer
PEEK	Polyetheretherketone
PDA	Polydopamine
FDA	Food and Drug Administration
FDM	Fused Deposition Modeling
SEM	Scanning Electron Microscope
TEM	Transmission Electron Microscope
EDS	Energy Dispersive X-Ray Spectroscope
DA	Dopamine

## References

[1] Liang H., Fu G., Liu J., Tang Y., Wang Y., Chen S., Zhang Y., Zhang C. (2022). A 3D-printed Sn-doped calcium phosphate scaffold for bone tissue engineering. Front. Mater..

[2] Pawelec K.M., Pawelec K.M., Planell J.A. (2019). 1—Introduction to the challenges of bone repair. Bone Repair Biomaterials.

[3] Peng K., Chen S., Senthooran V., Hu X., Qi Y., Zhang C., Wu L., Wang J. (2024). Microporous polylactic acid/chitin nanocrystals composite scaffolds using in-situ foaming 3D printing for bone tissue engineering. Int. J. Biol. Macromol..

[4] Pu F., Yu Y., Zhang Z., Wu W., Shao Z., Li C., Feng J., Xue L., Chen F. (2023). Research and Application of Medical Polyetheretherketone as Bone Repair Material. Macromol. Biosci..

[5] Zhang C., Ru Y., You J., Lin R., Chen S., Qi Y., Li D., Zhang C., Qiu Z. (2024). Antibacterial Properties of PCL@45s5 Composite Biomaterial Scaffolds Based on Additive Manufacturing. Polymers.

[6] Nasr Azadani M., Zahedi A., Bowoto O.K., Oladapo B.I. (2022). A review of current challenges and prospects of magnesium and its alloy for bone implant applications. Prog. Biomater..

[7] Lu Y., Cai W.-j., Ren Z., Han P. (2022). The Role of Staphylococcal Biofilm on the Surface of Implants in Orthopedic Infection. Microorganisms.

[8] Li P., Yin R., Cheng J., Lin J. (2023). Bacterial Biofilm Formation on Biomaterials and Approaches to Its Treatment and Prevention. Int. J. Mol. Sci..

[9] Panayotov I.V., Orti V., Cuisinier F., Yachouh J. (2016). Polyetheretherketone (PEEK) for medical applications. J. Mater. Sci. Mater. Med..

[10] Buck E., Li H., Cerruti M. (2020). Surface Modification Strategies to Improve the Osseointegration of Poly(etheretherketone) and Its Composites. Macromol. Biosci..

[11] Mishra S., Chowdhary R. (2019). PEEK materials as an alternative to titanium in dental implants: A systematic review. Clin. Implant Dent. Relat. Res..

[12] Koh Y.-G., Park K.-M., Lee J.-A., Nam J.-H., Lee H.-Y., Kang K.-T. (2019). Total knee arthroplasty application of polyetheretherketone and carbon-fiber-reinforced polyetheretherketone: A review. Mater. Sci. Eng. C.

[13] Xue N., Ding X., Huang R., Jiang R., Huang H., Pan X., Min W., Chen J., Duan J.-A., Liu P. (2022). Bone Tissue Engineering in the Treatment of Bone Defects. Pharmaceuticals.

[14] Ma H., Suonan A., Zhou J., Yuan Q., Liu L., Zhao X., Lou X., Yang C., Li D., Zhang Y.-g. (2021). PEEK (Polyether-ether-ketone) and its composite materials in orthopedic implantation. Arab. J. Chem..

[15] Shahrubudin N., Lee T.C., Ramlan R. (2019). An Overview on 3D Printing Technology: Technological, Materials, and Applications. Procedia Manuf..

[16] Hu X., Sansi Seukep A.M., Senthooran V., Wu L., Wang L., Zhang C., Wang J. (2023). Progress of Polymer-Based Dielectric Composites Prepared Using Fused Deposition Modeling 3D Printing. Nanomaterials.

[17] Bozkurt Y., Karayel E. (2021). 3D printing technology; methods, biomedical applications, future opportunities and trends. J. Mater. Res. Technol..

[18] Aimar A., Palermo A., Innocenti B. (2019). The Role of 3D Printing in Medical Applications: A State of the Art. J. Healthc. Eng..

[19] Wang X., Jiang M., Zhou Z., Gou J., Hui D. (2017). 3D printing of polymer matrix composites: A review and prospective. Compos. Part B Eng..

[20] Ma T., Zhang Y., Ruan K., Guo H., He M., Shi X., Guo Y., Kong J., Gu J. (2024). Advances in 3D printing for polymer composites: A review. InfoMat.

[21] Guo H., Thirunavukkarasu N., Mubarak S., Lin H., Zhang C., Li Y., Wu L. (2023). Preparation of Thermoplastic Polyurethane/Multi-Walled Carbon Nanotubes Composite Foam with High Resilience Performance via Fused Filament Fabrication and CO_2_ Foaming Technique. Polymers.

[22] Oladapo B.I., Zahedi S.A., Ismail S.O., Omigbodun F.T., Bowoto O.K., Olawumi M.A., Muhammad M.A. (2021). 3D printing of PEEK–cHAp scaffold for medical bone implant. Bio-Des. Manuf..

[23] Wang L., Yang C., Sun C., Yan X., He J., Shi C., Liu C., Li D., Jiang T., Huang L. (2022). Fused Deposition Modeling PEEK Implants for Personalized Surgical Application: From Clinical Need to Biofabrication. Int. J. Bioprinting.

[24] Toth J.M., Kurtz S.M. (2019). Chapter 8—Biocompatibility of PEEK Polymers. PEEK Biomaterials Handbook.

[25] Fu Q., Gabriel M., Schmidt F., Müller W.-D., Schwitalla A.D. (2021). The impact of different low-pressure plasma types on the physical, chemical and biological surface properties of PEEK. Dent. Mater..

[26] Oladapo B.I., Zahedi S.A., Ismail S.O., Omigbodun F.T. (2021). 3D printing of PEEK and its composite to increase biointerfaces as a biomedical material- A review. Colloids Surf. B Biointerfaces.

[27] McMullan R., Golbang A., Salma-Ancane K., Ward J., Rodzen K., Boyd A.R. (2025). Review of 3D Printing of Polyaryletherketone/Apatite Composites for Lattice Structures for Orthopedic Implants. Appl. Sci..

[28] Chen M., Luo C., Yuan Y., Zhou H., Li Z., Wang Q., Gong B., Li Z., Sun H. (2024). Modification of PEEK for implants: Strategies to improve mechanical, antibacterial, and osteogenic properties. Rev. Adv. Mater. Sci..

[29] Manzoor F., Golbang A., Jindal S., Dixon D., McIlhagger A., Harkin-Jones E., Crawford D., Mancuso E. (2021). 3D printed PEEK/HA composites for bone tissue engineering applications: Effect of material formulation on mechanical performance and bioactive potential. J. Mech. Behav. Biomed. Mater..

[30] Zheng J., Zhao H., Ouyang Z., Zhou X., Kang J., Yang C., Sun C., Xiong M., Fu M., Jin D. (2022). Additively-manufactured PEEK/HA porous scaffolds with excellent osteogenesis for bone tissue repairing. Compos. Part B Eng..

[31] Pan L., Guo H., Zhong L., Wang M., Xue P., Yuan X. (2021). Influence of surface-modified glass fibers on interfacial properties of GF/PEEK composites using molecular dynamics. Comput. Mater. Sci..

[32] Arslan E., Caglar I. (2025). Enhancing PEEK bond strength: The impact of chemical and mechanical surface modifications on surface characteristics and phase transformation. BMC Oral Health.

[33] Cardoso C., Matos J., Afonso C. (2025). Extraction of Marine Bioactive Compounds from Seaweed: Coupling Environmental Concerns and High Yields. Mar. Drugs.

[34] Bharathi D.S., Raja A.B., Nachimuthu S., Thangavel S., Kannan K., Shanmugan S., Tari V. (2025). Exploration of Bioactive Compounds, Antioxidant and Antibacterial Properties, and Their Potential Efficacy Against HT29 Cell Lines in Dictyota bartayresiana. Mar. Drugs.

[35] Liu D., Gao J., Wu X., Hao X., Hu W., Han L. (2025). Conductive Microneedles Loaded with Polyphenol-Engineered Exosomes Reshape Diabetic Neurovascular Niches for Chronic Wound Healing. Adv. Sci..

[36] Wang L.Q., Liu T.L., Liang P.H., Zhang S.H., Li T.S., Li Y.P., Liu G.X., Mao L., Luo X.N. (2020). Characterization of exosome-like vesicles derived from Taenia pisiformis cysticercus and their immunoregulatory role on macrophages. Parasites Vectors.

[37] Moosazadeh Moghaddam M., Fazel P., Fallah A., Sedighian H., Kachuei R., Behzadi E., Imani Fooladi A.A. (2023). Host and Pathogen-Directed Therapies against Microbial Infections Using Exosome- and Antimicrobial Peptide-derived Stem Cells with a Special look at Pulmonary Infections and Sepsis. Stem Cell Rev. Rep..

[38] Alexander M., Hu R., Runtsch M.C., Kagele D.A., Mosbruger T.L., Tolmachova T., Seabra M.C., Round J.L., Ward D.M., O’Connell R.M. (2015). Exosome-delivered microRNAs modulate the inflammatory response to endotoxin. Nat. Commun..

[39] Zhang W., Jiang X., Bao J., Wang Y., Liu H., Tang L. (2018). Exosomes in Pathogen Infections: A Bridge to Deliver Molecules and Link Functions. Front. Immunol..

[40] Casillo A., D’Amico R., Lanzetta R., Corsaro M.M. (2024). Marine Delivery Vehicles: Molecular Components and Applications of Bacterial Extracellular Vesicles. Mar. Drugs.

[41] Léguillier V., Heddi B., Vidic J. (2024). Recent Advances in Aptamer-Based Biosensors for Bacterial Detection. Biosensors.

[42] Ferreira I.M., de Sousa Lacerda C.M., dos Santos S.R., de Barros A.L.B., Fernandes S.O., Cardoso V.N., de Andrade A.S.R. (2017). Detection of bacterial infection by a technetium-99m-labeled peptidoglycan aptamer. Biomed. Pharmacother..

[43] Wu T., Wang H., Tian R., Guo S., Liao Y., Liu J., Ding B. (2023). A DNA Origami-based Bactericide for Efficient Healing of Infected Wounds. Angew. Chem. Int. Ed..

[44] Didarian R., Ozbek H.K., Ozalp V.C., Erel O., Yildirim-Tirgil N. (2025). Enhanced SELEX Platforms for Aptamer Selection with Improved Characteristics: A Review. Mol. Biotechnol..

[45] Hernandez J.L., Woodrow K.A. (2022). Medical Applications of Porous Biomaterials: Features of Porosity and Tissue-Specific Implications for Biocompatibility. Adv. Healthc. Mater..

[46] Sebastiani S., Buccino F., Qin Z., Vergani L.M. (2025). Structural influences on bone tissue engineering: A review and perspective. Matter.

[47] Zhang Y., Sun N., Zhu M., Qiu Q., Zhao P., Zheng C., Bai Q., Zeng Q., Lu T. (2022). The contribution of pore size and porosity of 3D printed porous titanium scaffolds to osteogenesis. Biomater. Adv..

[48] Lee H., Dellatore S.M., Miller W.M., Messersmith P.B. (2007). Mussel-Inspired Surface Chemistry for Multifunctional Coatings. Science.

[49] Chen J., Wu J., Mu J., Li L., Hu J., Lin H., Cao J., Gao J. (2023). An antioxidative sophora exosome-encapsulated hydrogel promotes spinal cord repair by regulating oxidative stress microenvironment. Nanomed. Nanotechnol. Biol. Med..

[50] Guo K., Wang Y., Feng Z.-X., Lin X.-Y., Wu Z.-R., Zhong X.-C., Zhuang Z.-M., Zhang T., Chen J., Tan W.-Q. (2024). Recent Development and Applications of Polydopamine in Tissue Repair and Regeneration Biomaterials. Int. J. Nanomed..

[51] Hu J., Ding Y., Tao B., Yuan Z., Yang Y., Xu K., Li X., Liu P., Cai K. (2022). Surface modification of titanium substrate via combining photothermal therapy and quorum-sensing-inhibition strategy for improving osseointegration and treating biofilm-associated bacterial infection. Bioact. Mater..

[52] Dong Q., Liang X., Chen F., Ke M., Yang X., Ai J., Cheng Q., Zhou Y., Chen Y. (2022). Injectable shape memory hydroxyethyl cellulose/soy protein isolate based composite sponge with antibacterial property for rapid noncompressible hemorrhage and prevention of wound infection. Int. J. Biol. Macromol..

[53] Guo Z., Zhang Z., Zhang N., Gao W., Li J., Pu Y., He B., Xie J. (2022). A Mg^2+^/polydopamine composite hydrogel for the acceleration of infected wound healing. Bioact. Mater..

[54] Youssef D.T.A., Alqarni A.S., Almohammadi A.M., Abujamel T., Shaala L.A. (2025). Marmaricines A-C: Antimicrobial Brominated Pyrrole Alkaloids from the Red Sea Marine Sponge Agelas sp. aff. marmarica. Mar. Drugs.

[55] Mohammadi P., Taghavi E., Foong S.Y., Rajaei A., Amiri H., de Tender C., Peng W., Lam S.S., Aghbashlo M., Rastegari H. (2023). Comparison of shrimp waste-derived chitosan produced through conventional and microwave-assisted extraction processes: Physicochemical properties and antibacterial activity assessment. Int. J. Biol. Macromol..

[56] El-Sheekh M.M., Yousuf W.E., Kenawy E.-R., Mohamed T.M. (2023). Biosynthesis of cellulose from Ulva lactuca, manufacture of nanocellulose and its application as antimicrobial polymer. Sci. Rep..

[57] Yang X., Hu Y., Zhou H., Lu N., Zhang M., Tang Z. (2025). Specific capture, detection, and killing of Enterococcus faecalis based on aptamer-modified peroxidase mimetic nanozymes. Chem. Eng. J..

[58] Sorgenfrei M., Hürlimann L.M., Remy M.M., Keller P.M., Seeger M.A. (2022). Biomolecules capturing live bacteria from clinical samples. Trends Biochem. Sci..

[59] Fatah S.A., Omer K.M. (2025). Aptamer-Modified MOFs (Aptamer@MOF) for Efficient Detection of Bacterial Pathogens: A Review. ACS Appl. Mater. Interfaces.

