# Supplementary material for: Engineering Self-Assembled PEEK Scaffolds with Marine-Derived Exosomes and Bacteria-Targeting Aptamers for Enhanced Antibacterial Functions
Source: J Funct Biomater. 2025 Dec 30;17(1):23. doi: 10.3390/jfb17010023 (PMC12842172; doi:10.3390/jfb17010023)
Supplement: Supplementary file 1 [file jfb-17-00023-s001.zip › jfb-4027339-supplementary.pdf]

Table S1. Measurement results of weight, diameter, and height of composite scaffolds

		**1**	**2**	**3**	**Average**	**σ**
PEEK	Weight (g)	0.3761	0.3753	0.3839	0.3784	0.0039
	Diameter (mm)	14.88	14.96	15.00	14.95	0.0500
	Height(mm)	4.98	4.92	5.12	5.01	0.0839
PEEK-PDA	Weight (g)	0.3877	0.3846	0.3712	0.3812	0.0072
	Diameter (mm)	15.22	14.76	15.18	15.05	0.2081
	Height(mm)	5.14	4.86	5.20	5.07	0.1482

Table S2. Size of the antibacterial zone of the scaffolds*

	Escherichia coli				***Staphylococcus aureus***			
	PEEK	50	100	200	PEEK	50	100	200
Parallel 1(mm)	ND	15.64	16.34	17.84	ND	15.76	16.48	17.72
Parallel 2(mm)	ND	15.70	16.48	17.78	ND	15.68	16.64	17.82
Parallel 3(mm)	ND	15.74	16.54	17.70	ND	15.80	16.28	17.64
Average(mm)	ND	15.69	16.45	17.77	ND	15.75	16.47	17.73

*50, 100, 200 and ND represent the experimental groups of Apt modified scaffolds with concentrations of PEEK-PDA-EXOs-Apt50nM, PEEK-PDA-EXOs-Apt100nM, PEEK-PDA-EXOs-Apt200nM, and not detected, respectively.
